# Dynamic Surveillance of Minimal Residual Disease via a Tumor-Informed Circulating Tumor DNA Assay for Outcome Prediction in Small-Cell Lung Cancer: An Exploratory Pilot Study

**DOI:** 10.3390/biomedicines14050972

**Published:** 2026-04-23

**Authors:** Qiuyi Zhang, Die Dai, Yikun Yang, Lihong Guo, Jiesheng Su, Shiqi Lyu, Suni Huang, Meng Zhang, Jianhua Chang

**Affiliations:** 1 National Cancer Center/National Clinical Research Center for Cancer/Cancer Hospital & Shenzhen Hospital, Chinese Academy of Medical Sciences and Peking Union Medical College, Shenzhen 518116, China; zqystephy2026@163.com (Q.Z.); mib_yyk@hotmail.com (Y.Y.); m15970030032@163.com (L.G.); sujiesheng@163.com (J.S.); 13528440145@163.com (S.L.); 13428905757@163.com (S.H.); 2 BGI Genomics, Shenzhen 518083, Chinazhangmeng3@bgi.com (M.Z.)

**Keywords:** small-cell lung cancer, circulating tumor DNA, minimal residual disease, liquid biopsy, tumor-informed

## Abstract

**Background**: Small-cell lung cancer (SCLC) represents an aggressive malignancy associated with a poor prognosis, underscoring the critical demand for enhanced monitoring methodologies. Circulating tumor DNA (ctDNA) constitutes a promising non-invasive biomarker; however, reports employing highly sensitive tumor-informed assays in SCLC remain scarce. This investigation aimed to assess the clinical utility of a personalized ctDNA monitoring strategy for predicting therapeutic outcomes and resistance in SCLC patients. **Methods**: This prospective observational study enrolled patients diagnosed with unresectable SCLC. Whole exome sequencing was conducted on baseline tumor specimens to design customized 16-plex multiplex polymerase chain reaction (PCR) panels. Serial blood samples were obtained at baseline, at six-week intervals during treatment, and upon disease progression. Detection of ctDNA-based minimal residual disease (MRD) was performed using a tumor-informed assay (Huajianwei^®^ bespoke MRD) with ultra-deep sequencing. **Results**: Among seven evaluable patients, the baseline ctDNA-MRD positivity rate was 100%. A significant positive correlation was observed between the baseline ctDNA levels and radiographic tumor burden (r = 0.821, 95% confidence interval [CI] 0.179–0.973, *p* = 0.034). Longitudinal analysis indicated that patients exhibiting an early decline in MRD levels demonstrated a non-significant trend toward superior progression-free survival (PFS) compared to those with an MRD increase. Though this between-group difference did not reach conventional statistical significance, it represented a trend-level finding (*p* = 0.0665, hazard ratio [HR] = 0.24, 95% CI: 0.02–3.19), with no definitive prognostic association confirmed in this pilot cohort. Notably, an elevation in MRD preceded radiographic progression by as much as 135 days in certain instances. **Conclusions**: This study shows that dynamic tumor-informed ctDNA-based MRD monitoring reflects tumor burden changes and may correlate with clinical outcomes in SCLC, supporting its potential to guide personalized treatment and facilitate earlier therapeutic interventions compared to conventional imaging techniques. Prospective multicenter validation is needed to confirm its clinical utility.

## 1. Introduction

Lung cancer remains the foremost cause of cancer-associated mortality globally [1]. Primary lung cancer can be categorized into two major pathological subtypes: non-small cell lung cancer (NSCLC) and small-cell lung cancer (SCLC). Although SCLC constitutes only 15% to 20% of all primary lung cancer cases [2], it is distinguished by a high degree of malignancy, poor differentiation, and rapid proliferation, contributing to an exceptionally poor prognosis with a five-year survival rate below 5% [3,4]. Given its aggressive characteristics, there exists a pressing and substantial clinical requirement for effective strategies for early disease detection, real-time assessment of the therapeutic response, and accurate prognostic forecasting.

Circulating tumor DNA (ctDNA) has emerged as a precise non-invasive predictive biomarker with considerable potential across the spectrum of cancer management, encompassing early screening, diagnosis, prognostic assessment, and treatment monitoring [5,6,7]. In SCLC, dynamic ctDNA changes serve as a sensitive marker of treatment response and prognosis [8,9]. ctDNA status also helps identify patients likely to benefit from immunotherapy [10]. Molecular response—defined as the clearance of mutations and copy number variations—predicts clinical outcomes approximately four weeks before conventional imaging and is associated with significantly improved progression-free survival (PFS) and overall survival (OS) [11]. In contrast, high baseline ctDNA levels and persistent ctDNA positivity after consolidation immunotherapy correlate with poor prognosis [10,11,12,13]. Extensive evidence supports minimal residual disease (MRD) as a key predictor of outcomes in locally advanced or advanced lung cancer [14,15], with next-generation sequencing (NGS)-based ctDNA detection serving as the primary monitoring method [16].

In solid tumors, two principal strategies are employed for MRD detection: tumor-informed and tumor-agnostic analysis. The tumor-informed approach represents a highly sensitive and specific technique [17,18]. It involves performing NGS on blood samples using a predefined panel of oncogenic mutations or employing machine learning algorithms to identify potential tumor-derived ctDNA variants. Furthermore, by conducting comparative analyses between tumor and normal tissues and utilizing small targeted panels (typically spanning a few kilobases) sequenced to ultra-high depth (often exceeding 100,000× coverage) [19], the tumor-informed method effectively distinguishes the ctDNA signals of tumor origin from background noise, such as clonal hematopoiesis or germline variants. This significantly enhances both the sensitivity and specificity of detection. In contrast, the tumor-agnostic method, with relatively lower sensitivity, performs NGS directly on blood samples, utilizing pre-defined oncogene mutation panels or machine learning algorithms to filter for potential tumor-derived ctDNA variants [17]. Because tumor-agnostic panels are so broad, they may lack the depth of coverage needed to detect alterations relevant to a given patient.

Although prior studies have demonstrated correlations between ctDNA levels and treatment efficacy or prognosis in SCLC patients [9,11,13], reports utilizing the highly sensitive tumor-informed methodology remain limited. In this study, we performed whole exome sequencing (WES) on SCLC tissue samples to design and synthesize customized panels. This enabled the personalized and dynamic monitoring of MRD in peripheral blood samples throughout the treatment course. The objective was to investigate the clinical utility of this customized plasma ctDNA-based MRD monitoring approach in SCLC patients and its significance in predicting therapeutic outcomes and the emergence of resistance.

## 2. Materials and Methods

### 2.1. Study Design and Patients

**Figure 1:**
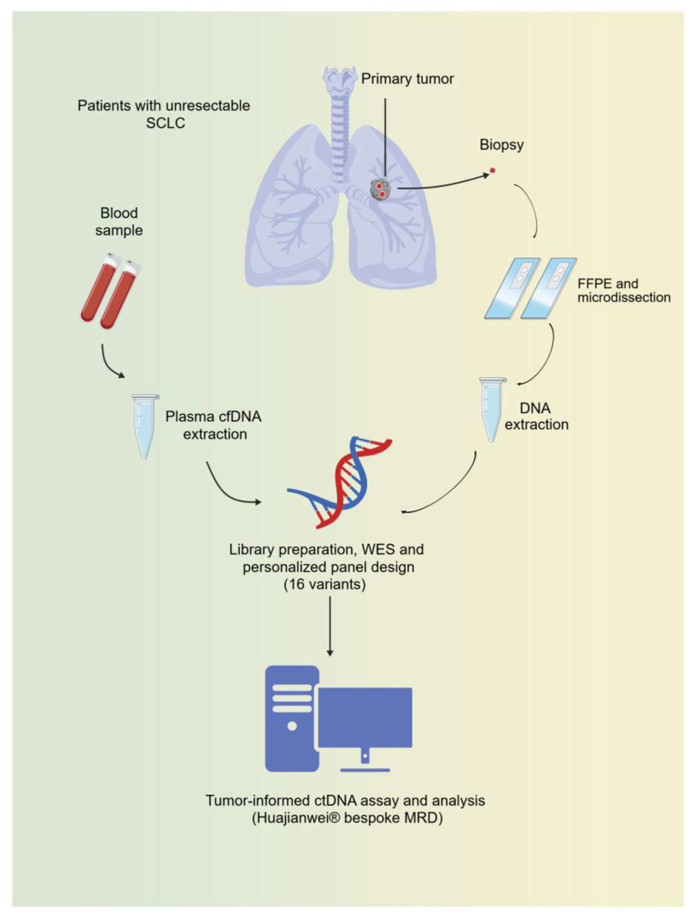
The flowchart of this study. Whole exome sequencing was conducted on baseline tumor specimens from 20 participants. Ultimately, personalized panels were successfully customized for seven patients, due to insufficient tumor specimens for the other participants. Serial peripheral blood samples from the enrolled patients were collected at baseline, every six weeks, and upon disease progression. Detection of ctDNA-based MRD was performed via a tumor-informed assay with ultra-deep sequencing. The trends in these measurements were then analyzed in relation to clinical efficacy and outcomes. SCLC, small-cell lung cancer; cfDNA, cell-free DNA; FFPE, formalin-fixed paraffin-embedded; WES, whole exome sequencing. MRD, minimal residual disease.

This was a prospective, observational, single-cohort study. The research flowchart is shown in Figure 1 (created with BioGDP.com, accessed on 20 April 2026 [20]). The inclusion criteria were as follows: (1) age ≥ 18 years; (2) histopathologically confirmed unresectable SCLC; (3) a life expectancy exceeding three months; (4) availability of sufficient tumor tissue for WES; (5) ability to provide adequate peripheral blood samples for ctDNA-based MRD detection during treatment; and (6) presence of at least one measurable lesion according to Response Evaluation Criteria in Solid Tumors (RECIST) version 1.1 [21]. The primary endpoint was the correlation between ctDNA-based MRD status and radiologically assessed PFS.

**Figure 2:**
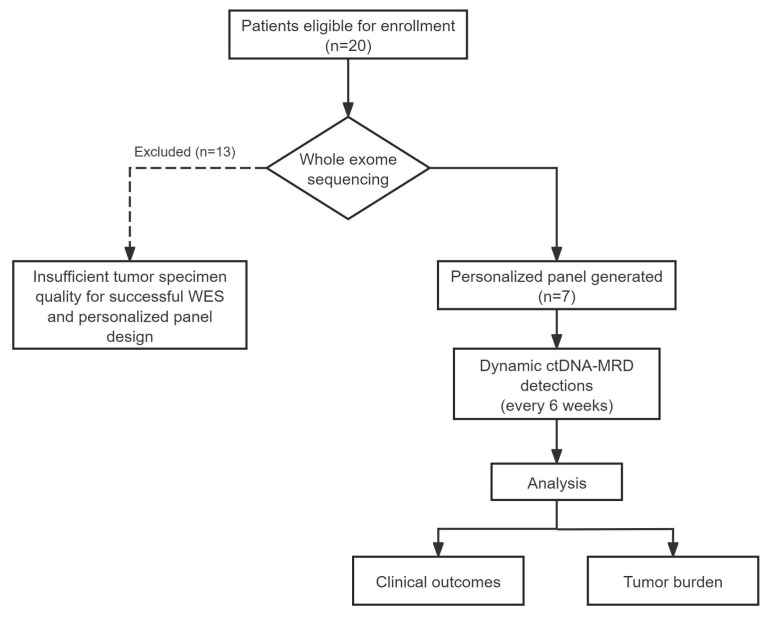
The flow diagram for patient enrollment. Twenty patients were enrolled in this study. Ultimately, personalized panels were successfully customized only for seven patients due to insufficient tumor specimens for other patients. WES, whole exome sequencing; ctDNA, circulating tumor DNA; MRD, minimal residual disease.

A total of 20 patients were enrolled in this study. However, due to the insufficient tumor specimen quality for successful WES and personalized panel design, personalized ctDNA panels were successfully generated only for seven individuals. Figure 2 presents the flow diagram for patient enrollment.

### 2.2. Sample Collection

Baseline tumor formalin-fixed paraffin-embedded (FFPE) samples were collected (consisting of 15–20 sections, each 5 µm thick). A total of 20 mL of peripheral venous blood was collected at baseline, every six weeks during treatment, and at the time of disease progression. These samples were transported to the BGI Tianjin Specimen Center within 72 h of collection.

### 2.3. Efficacy Evaluation

Computed tomography (CT) and/or magnetic resonance imaging (MRI) was performed every six weeks (±seven days) during treatment. The therapeutic efficacy was evaluated according to RECIST version 1.1. PFS was defined as the time from treatment initiation to the first documentation of objective tumor progression or death from any cause, whichever occurred first. Patients who were lost to follow-up were censored at the date of their last known contact. For those who remained alive at the study cutoff date, survival data were censored on that date.

### 2.4. Extraction of Cell-Free DNA (cfDNA)

cfDNA, including ctDNA, was isolated from human plasma using the QIAamp Circulating Nucleic Acid Kit (Qiagen, Hilden, Germany) according to the manufacturer’s instructions. Briefly, the plasma samples were subjected to enzymatic lysis with QIAGEN Proteinase K and Buffer ACL (supplemented with carrier RNA) at 60 °C for 30 min to ensure the complete release of nucleic acids from proteins and vesicles. Binding conditions were adjusted by the addition of Buffer ACB, and the resulting lysate was drawn through a QIAamp Mini column (Qiagen, Hilden, Germany) using the QIAvac 24 Plus vacuum manifold (Qiagen, Hilden, Germany). The silica membrane was sequentially washed with Buffers ACW1, ACW2, and 96–100% ethanol to efficiently remove the residual contaminants and inhibitors. Finally, the purified nucleic acids were eluted in the Buffer AVE. The extracted DNA was either used immediately for downstream applications or stored at −20 °C for long-term stability.

### 2.5. WES at Baseline

Fresh tumor tissue samples were FFPE, stained with hematoxylin and eosin (H&E), and subjected to pathological review to confirm a tumor content of at least 30%. Genomic DNA (gDNA) was extracted from the tumor tissue using the QIAamp DNA FFPE Tissue Kit (Qiagen, Hilden, Germany) and from matched whole blood samples using the MagPure Buffy Coat DNA Midi KF Kit (Magen, Guangzhou, China). The extracted DNA was then quantified by Qubit 3.0 using the dsDNA HS Assay Kit (ThermoFisher Scientific, Singapore).

For library preparation, approximately 400 ng of tumor gDNA and 200 ng of germline DNA underwent fragmentation, end repair, 3′-adenylation, and adapter ligation. The resulting libraries were pooled and hybridized to the Quanxi^®^ pan-cancer whole exome panel. Sequencing was performed on the MGISEQ-2000 platform (MGI, Shenzhen, China), achieving a mean coverage depth of 500× for tumor samples and 200× for normal controls, which was set to ensure the accuracy and confidence of somatic variant calling for subsequent personalized panel design. In addition to identifying single nucleotide variants (SNVs), insertions/deletions (indels), copy number variations (CNVs), and rearrangements, the analysis included evaluations of the tumor mutational burden (TMB) and microsatellite instability (MSI). To facilitate personalized monitoring, sequencing data were processed through the Signatera™ WES pipeline to select 16 prioritized SNVs per patient. Based on these variants, 16 specific multiplex PCR primer pairs were optimized, designed, and synthesized at BGI Tech Solutions for subsequent ctDNA detection.

### 2.6. Personalized Tumor-Informed ctDNA Detection

At each monitoring time point, 20 mL of peripheral blood was collected in cfDNA BCT^®^ tubes (Streck, La Vista, NE, USA) and processed using the Huajianwei^®^ bespoke MRD assay (BGI Genomics, Shenzhen, China), a tumor-informed approach based on the Signatera™ platform as previously described [22,23]. cfDNA was extracted from a median plasma volume of 8 mL using the QIAamp Circulating Nucleic Acid Kit (Qiagen, Hilden, Germany). Subsequently, 10–66 ng of cfDNA was used for library preparation with patient-specific primer sets targeting the 16 patient-specific SNVs pre-identified via WES, ensuring the robustness of longitudinal monitoring. In this method, the 16 prioritized SNVs previously identified via WES were targeted. Personalized 16-plex primer pairs were employed to amplify the universal cfDNA libraries, which were then sequenced on the MGISEQ-2000 platform to a median depth exceeding 110,000× per amplicon, to provide sufficient analytical sensitivity required for the detection of low-abundance ctDNA fragments in plasma samples. Data analysis was performed using the Huajianwei^®^ plasma pipeline (BGI Genomics, Shenzhen, China, pipeline Version: 3.0.0.5-BGI-SNAPSHOT-1605915), and MRD positivity was defined by the detection of at least two patient-specific variants. The ctDNA burden was quantified as the mean tumor molecules per milliliter of plasma (MTM/mL). The assay demonstrates a sensitivity of >95% for detecting variants at a 0.03% variant allele frequency (VAF). A “meaningful change” was defined pragmatically as a sustained increase or decrease in the aggregate VAF of tracked mutations by a factor of >2-fold from baseline, observed over at least two consecutive time points to ensure trend consistency.

### 2.7. Statistical Analysis

Survival analysis was conducted using the Kaplan–Meier method, and differences between groups were compared using the log-rank test. The correlation between ctDNA levels and the sum of diameters of target lesions was analyzed using Spearman’s rank correlation coefficient. All statistical tests were two-sided, and a *p*-value < 0.05 was considered statistically significant. Given the exploratory and pilot nature of this study, no formal adjustments for multiple comparisons were performed. Data analysis was performed using R software (version 4.1). In this clinical observational study, biological replicates are represented by the individual patients.

## 3. Results

### 3.1. Baseline Demographics and Clinical Attributes

Twenty patients were enrolled in this study. However, due to the insufficient tumor specimen quality for successful WES and personalized panel design, personalized ctDNA panels were successfully generated for only seven individuals (Figure 2). The clinical data are summarized in Table 1. The cohort was predominantly male, with a median age of 60 years (range: 58 to 74 years). All participants had a documented smoking history, with substantial cumulative exposure ranging from 15 to 122 pack-years. Regarding disease severity at baseline, two patients presented with limited-stage disease, while the remaining five had extensive-stage disease. The majority of the cohort was diagnosed with pure SCLC, and one patient was diagnosed with combined SCLC (C-SCLC).

The administered treatment varied across different lines of therapy. Six patients received platinum-based combination chemotherapy, frequently combined with thoracic radiotherapy, programmed cell death ligand-1 (PD-L1) inhibitors, or PD-1 inhibitors. One patient was administered anti-angiogenesis monotherapy as a fifth-line treatment. The clinical outcomes exhibited variability, with PFS durations spanning from 1.0 to 19.3 months. The best overall responses comprised partial response (PR) in two patients, stable disease (SD) in three patients, and progressive disease (PD) in two patients. TMB also demonstrated considerable heterogeneity, ranging from 4.81 to 61.8 mutations per megabase. The PD-L1 expression levels were predominantly low or not assessed, with documented tumor proportion score (TPS) of either 0% or less than 1%.

### 3.2. Clinical Relevance of Baseline WES and ctDNA Analysis

**Figure 3:**
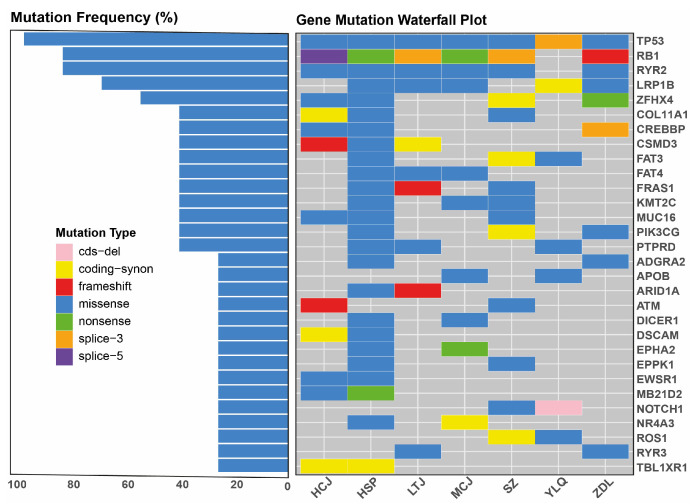
Genomic profile of the enrolled patients by WES (n = 7). *TP53* and *RB1* were the most commonly altered genes, present in 100% and 85.7% of the cohort, respectively. Other frequently observed mutations included *RYR2*, *LRP1B*, and *ZFHX4*. WES, whole exome sequencing.

WES was conducted to delineate the baseline genomic profile of the enrolled patients. The evaluation of the mutation frequency identified *TP53* and *RB1* as the most commonly altered genes, present in 100% and 85.7% of the cohort, respectively (Figure 3). Other frequently observed mutations included *RYR2* (approximately 80%), *LRP1B* (approximately 70%), and *ZFHX4* (approximately 60%). The gene mutation waterfall plot revealed a predominance of missense mutations among the detected genes, accompanied by frameshift, nonsense, and splice-site variants. Notably, the baseline positivity rate for ctDNA-based MRD detection was 100%, as ctDNA was successfully detected in all seven patients prior to the commencement of study treatment.

**Figure 4:**
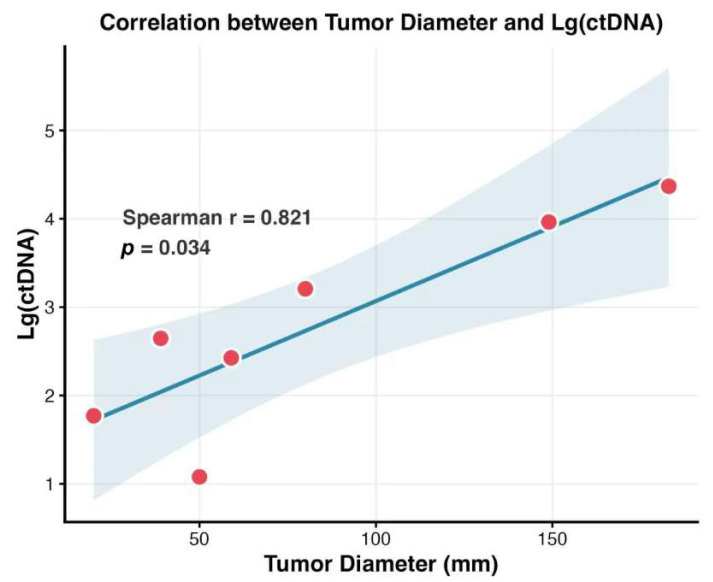
Spearman’s rank correlation between baseline ctDNA levels and the longest diameters of target lesions. Red dots represent individual data points. The solid blue line indicates the linear regression trend, and the light blue shaded area represents the 95% confidence interval. ctDNA, circulating tumor DNA.

Beyond genomic characterization, we further examined the association between ctDNA levels and physical tumor burden. Spearman’s rank correlation analysis indicated a significant positive linear relationship between the baseline ctDNA quantification and the sum of the longest diameters of target lesions, producing a correlation coefficient of 0.821 (95% confidence interval [CI] 0.179–0.973, *p* = 0.034). These results, depicted in a scatter plot (Figure 4), imply that ctDNA can effectively function as a molecular proxy for the overall tumor volume.

### 3.3. Longitudinal Assessment of ctDNA-MRD During Treatment

**Figure 5:**
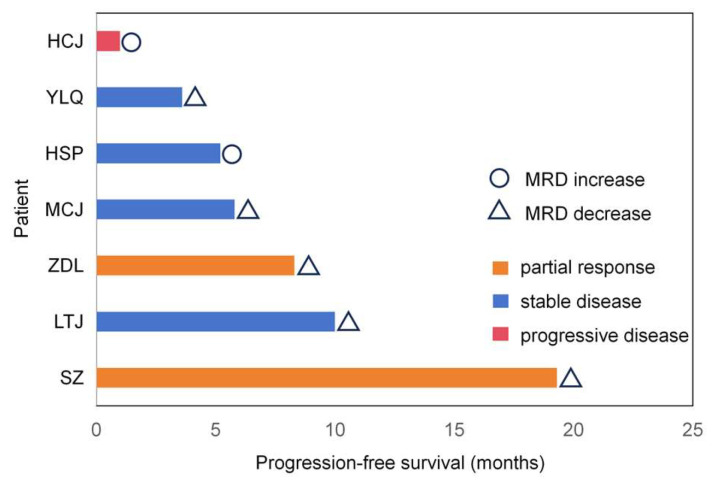
Swimmer plot of the participants. MRD, minimal residual disease.

**Figure 6:**
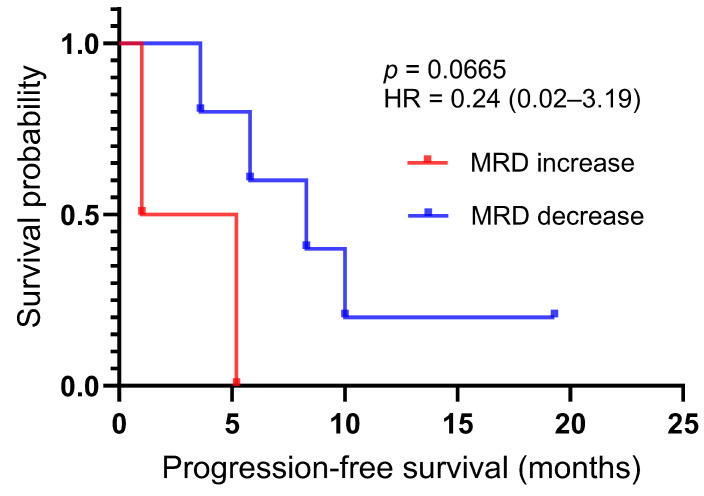
Kaplan–Meier survival curves of the MRD increase and decrease cohorts. Patients in the MRD-decrease exhibited a non-significant tendency toward longer PFS. MRD, minimal residual disease; PFS, progression-free survival.

Longitudinal monitoring of MRD was performed for all seven patients to assess the predictive utility of ctDNA dynamics. A total of 23 blood samples were collected for analysis. Based on the MRD trends observed at the second monitoring time point (the first re-staging scan six weeks after treatment initiation), patients were categorized into two distinct groups: the MRD-decrease group (n = 5) and the MRD-increase group (n = 2), as shown in the swimmer plot (Figure 5). Patients in the MRD-decrease group achieved either PR or SD as their best response, with a tendency toward longer PFS, extending up to 19.3 months; due to the limited cohort size, a *p*-value of 0.0665 was obtained, which did not reach the conventional two-sided threshold for statistical significance (*p* < 0.05). The hazard ratio (HR) of 0.24 (95% CI: 0.02–3.19) only supports a trend toward a reduced risk of disease progression in patients with declining ctDNA levels, rather than a definitive prognostic association (Figure 6). Remarkably, one patient attained sustained MRD clearance after three cycles of sequential chemoradiotherapy; this individual remains alive with an OS currently surpassing 30 months. In contrast, the two patients demonstrating an early increase in MRD experienced rapid disease progression, with PFS durations of only 5.2 and 1.0 months, respectively. These findings underscore that early on-treatment ctDNA-MRD kinetics may constitute a crucial indicator of therapeutic response and long-term prognosis in SCLC patients. Moreover, our longitudinal data suggest that MRD elevation can act as an early warning signal, preceding radiographic disease progression by up to 135 days. However, the limited cohort size reduces the statistical power and precludes definitive conclusions, and our findings should be interpreted as preliminary and hypothesis-generating.

## 4. Discussion

This study represents an innovative application of a tumor-informed methodology for dynamic MRD monitoring in SCLC patients, offering a preliminary investigation into the relationship between ctDNA-MRD kinetics and clinical efficacy. Despite the inherent constraints of a limited sample size, our findings reveal a distinct clinical signal: patients achieving a significant reduction or clearance of MRD during treatment demonstrated a trend toward superior PFS. This was exemplified most strikingly by a single patient who achieved MRD clearance after only three cycles of chemotherapy and subsequently attained an OS exceeding 30 months. Molecular response usually refers to the therapeutic response at the molecular biology level; that is, the therapeutic effect is evaluated through changes in molecular markers (such as the level of ctDNA). This case strongly suggests that MRD clearance could serve as a surrogate marker for “molecular complete response”, potentially offering prognostic insights as significant as radiographic complete response. Furthermore, the positive correlation identified between ctDNA and tumor burden provides empirical support for ctDNA as a quantitative surrogate in SCLC management. However, we note that while the direction of correlation aligns with expectations from other cancers, its magnitude and reliability in SCLC require confirmation in larger studies, acknowledging factors such as the tumor stage and various treatment lines as potential sources of variability.

From a mechanistic perspective, the dynamic fluctuations in MRD likely reflect the differential sensitivity of specific tumor clones to cytotoxic agents. Given that SCLC is characterized by high proliferative activity and a propensity for early systemic dissemination, traditional imaging often fails to capture micro-residual disease or occult metastases. As genetic fragments released into the bloodstream via apoptosis or necrosis, ctDNA may serve as a more sensitive indicator, capable of reflecting the evolution of the total tumor burden earlier and more accurately than conventional methods. The observed correlation between ctDNA levels and the sum of target lesion diameters validates this biological rationale and provides a theoretical foundation for future trials exploring whether the ctDNA–MRD status could justify extending intervals between radiographic evaluations.

When compared with the existing literature, our results align with several prospective cohorts in SCLC. Previous research by Chaudhuri et al. noted that the post-treatment ctDNA status was highly associated with the recurrence risk in limited-stage small-cell lung cancer (LS-SCLC) [6]. Similarly, research by Moding et al. confirmed that the long-term survival rates for patients achieving MRD clearance were significantly higher than for those who did not [24]. Studies have demonstrated a correlation between tumor volume and the VAF in ctDNA [25,26]. Our research also yielded similar findings. By replicating these findings, our study suggests that the prognostic stratification value of dynamic MRD monitoring may be a universal feature that transcends specific disease stages and may be applicable across the SCLC spectrum.

We must acknowledge that the small sample size is the primary limitation of this study. Patient attrition, particularly due to tissue availability, could introduce a selection bias (e.g., potentially favoring patients with larger initial tumor biopsies) and limits the generalizability of the findings. Additionally, the varied treatment regimens and lines of therapy among the seven patients represent a significant confounding factor, making it difficult to disentangle the effect of ctDNA dynamics from the differential efficacy of the treatments themselves. Due to the very small number of patients in each treatment category, meaningful subgroup or sensitivity analyses were not feasible. Lastly, the optimal timing and thresholds for MRD monitoring remain unstandardized; the trend-based classification at the first six weeks used in this study remains exploratory, and future studies should aim to define and validate standardized clinically actionable thresholds for molecular response in SCLC. Despite these limitations, our study provides preliminary evidence that ctDNA dynamics may correlate with clinical outcomes in this challenging patient population. These hypothesis-generating findings warrant validation in larger prospective cohorts.

Future research should pivot toward several key directions to build upon these preliminary findings. First, the initiation of prospective multicenter cohorts is necessary to systematically evaluate the predictive efficacy of MRD kinetics at various clinical milestones. Second, there is a clear need to explore MRD-driven adaptive therapeutic strategies, such as treatment de-escalation for those with durable MRD clearance or treatment intensification for those with persistent molecular positivity. Finally, researchers should focus on integrated modeling that combines genomic features with MRD dynamics to enhance personalized prognostic precision. It is worth noting that while the prospects are broad, ctDNA detection in SCLC still faces challenges. For example, *TP53* mutations can be detected in the cfDNA of non-cancerous populations due to clonal hematopoiesis of indeterminate potential (CHIP) [27,28], limiting its use in early screening. CHIP represents a natural consequence of aging, marked by the gradual buildup of somatic mutations in hematopoietic stem cells that drive their clonal expansion. Since cfDNA largely derives from these cells, CHIP poses a major confounding factor in the interpretation of ctDNA results. By using a tumor-informed approach, where variants are first identified in the tumor tissue and their presence in matched white blood cell DNA is excluded, we specifically design panels to track tumor-derived mutations, thereby largely mitigating the risk of false-positive signals from CHIP. Additionally, in heavily pre-treated patients, ctDNA dynamics may show a poor correlation with radiographic responses under certain combination therapies [29], suggesting its value may vary by treatment regimen and clinical context. Future studies are needed to further standardize detection protocols and validate clinical utility in large-scale trials across diverse therapeutic scenarios.

## 5. Conclusions

This investigation demonstrates that dynamic MRD monitoring via ctDNA effectively mirrors fluctuations in tumor burden and may be associated with clinical outcomes in SCLC patients. These findings support the potential of ctDNA in enhancing precision and personalized treatment strategies for SCLC. However, validation in larger, prospective multicenter cohorts is critically needed to determine the true clinical utility of this approach in SCLC.

## Figures and Tables

**Table 1:** Patient characteristics.

Patient	Age	Sex	Smoking, Pack-Years	Pathological Diagnosis	Tumor Stage	Treatment	Lines	PFS (Months)	Tumor Response	TMB (Muts/Mb)	**PD-L1** **(TPS)**
LTJ	58	Male	80	SCLC	LS	platinum-etoposide with thoracic radiotherapy	1st	10.0	SD	7.97	NA
MCJ	60	Male	40	SCLC	ES	platinum-etoposide with PD-L1 inhibitor	1st	5.8	SD	5.19	0%
ZDL	59	Male	40	SCLC	ES	platinum-etoposide with thoracic radiotherapy	2nd	8.3	PR	5.01	NA
HSP	60	Male	122	SCLC	ES	platinum-etoposide with PD-1 inhibitor	3rd	5.2	SD	61.8	NA
YLQ	61	Male	60	SCLC	ES	anlotinib	5th	3.6	PD	4.81	<1%
HCJ	60	Male	15	C-SCLC	ES	platinum-etoposide	3rd	1.0	PD	8.25	<1%
SZ	74	Male	30	SCLC	LS	platinum-etoposide with thoracic radiotherapy	1st	19.3	PR	11.17	NA

Abbreviations: SCLC, small-cell lung cancer; C-SCLC, combined small-cell lung cancer; LS, limited stage; ES, extensive stage; PFS, progression-free survival; SD, stable disease; PD, progressive disease; PR, partial response. TMB, tumor mutational burden; PD-(L) 1, programmed cell death (ligand)-1; TPS, tumor proportion score; NA, not available.

## Data Availability

The original contributions presented in this study are included in the article/Supplementary Materials. Further inquiries can be directed to the corresponding author.

## Supplementary Materials

The following supporting information can be downloaded at: https://www.mdpi.com/article/10.3390/biomedicines14050972/s1, Table S1: Raw data of Spearman’s rank correlation coefficient; Table S2: Data of the PFS and ctDNA-MRD levels. Table S3: Raw data of WES.

## Abbreviations

The following abbreviations are used in this manuscript:

SCLC	Small-cell lung cancer
ctDNA	circulating tumor DNA
PFS	progression-free survival
OS	overall survival
LS-SCLC	limited-stage small-cell lung cancer
MRD	minimal residual disease
NGS	next-generation sequencing
WES	whole exome sequencing
RECIST	Response Evaluation Criteria in Solid Tumors
FFPE	formalin-fixed paraffin-embedded
CT	computed tomography
MRI	magnetic resonance imaging
cfDNA	cell-free DNA
H&E	hematoxylin and eosin
gDNA	genomic DNA
SNVs	single nucleotide variants
indels	insertions/deletions
CNVs	copy number variations
TMB	tumor mutational burden
MSI	microsatellite instability
MTM/mL	mean tumor molecules per milliliter
VAF	variant allele frequency
PD-(L)1	programmed cell death (ligand)-1
PR	partial response
SD	stable disease
PD	progressive disease
TPS	tumor proportion score
HR	hazard ratio
NSCLC	non-small-cell lung cancer
CI	confidence interval
PCR	polymerase chain reaction
C-SCLC	combined small-cell lung cancer
CHIP	clonal hematopoiesis of indeterminate potential
